# An alternative technical marker set for the pelvis is more repeatable than the standard pelvic marker set

**DOI:** 10.1016/j.gaitpost.2013.05.019

**Published:** 2013-09

**Authors:** Maedeh Borhani, Alison H. McGregor, Anthony M.J. Bull

**Affiliations:** a Department of Bioengineering and Surgery and Cancer, Royal School of Mines, South Kensington Campus, Imperial College London, London SW7 2AZ, United Kingdom; b Faculty of Medicine, Department of Surgery and Cancer, Charing Cross Hospital, Charing Cross Campus, Imperial College London, United Kingdom; c Department of Bioengineering, 3.11, Royal School of Mines, South Kensington Campus, Imperial College London, United Kingdom

**Keywords:** Pelvic kinematics, Motion analysis, Marker set, Repeatability, Soft tissue artefact

## Abstract

Multiple marker sets and models are currently available for assessing pelvic kinematics in gait. Despite the presence of a variety models, there are still debates on their reliability and consistency, and consequently there is no clearly defined standard. Two marker sets were evaluated in this study: the ‘Traditional’ where markers are placed at the anterior and posterior superior iliac spines (ASISs, PSISs); and the ‘Cluster’, where a cluster of three orthogonal markers fixed on a rigid based is attached to the sacrum. The two sets were compared with respect to intra and inter session standard deviations of maximum pelvic tilt, obliquity and rotation angles. The repeatability between and within sessions was measured using coefficient of multiple correlation (CMC). Also the similarity between the two sets was assessed using inter-protocol CMC (ipCMC). Both data sets generated showed high within and between session repeatability in the sagittal plane (CMC > 0.80), although the Cluster method showed higher repeatability than that of the Traditional method in non-sagittal plane motion for both within and between sessions. The authors are not aware of other studies reporting the differences in intra and inter session variability and repeatability values for different body mass index categories such as overweight and obese subjects with relatively large sample size. Hence the Cluster method overcomes a number of theoretical and experimental limitations such as minimising the marker occlusion and is a reliable alternative to the Traditional (the standard) marker set.

## Highlights

• Pelvic kinematics was studied using two sets of markers.
• The standard set was compared to a cluster consists of three orthogonal markers.
• There were no significant differences between the two methods for normal subjects.
• The Cluster set showed higher repeatability for overweight and obese subjects.
• The Cluster set showed less variability for overweight and obese subjects.

## 1 Introduction

**Fig. 1:**
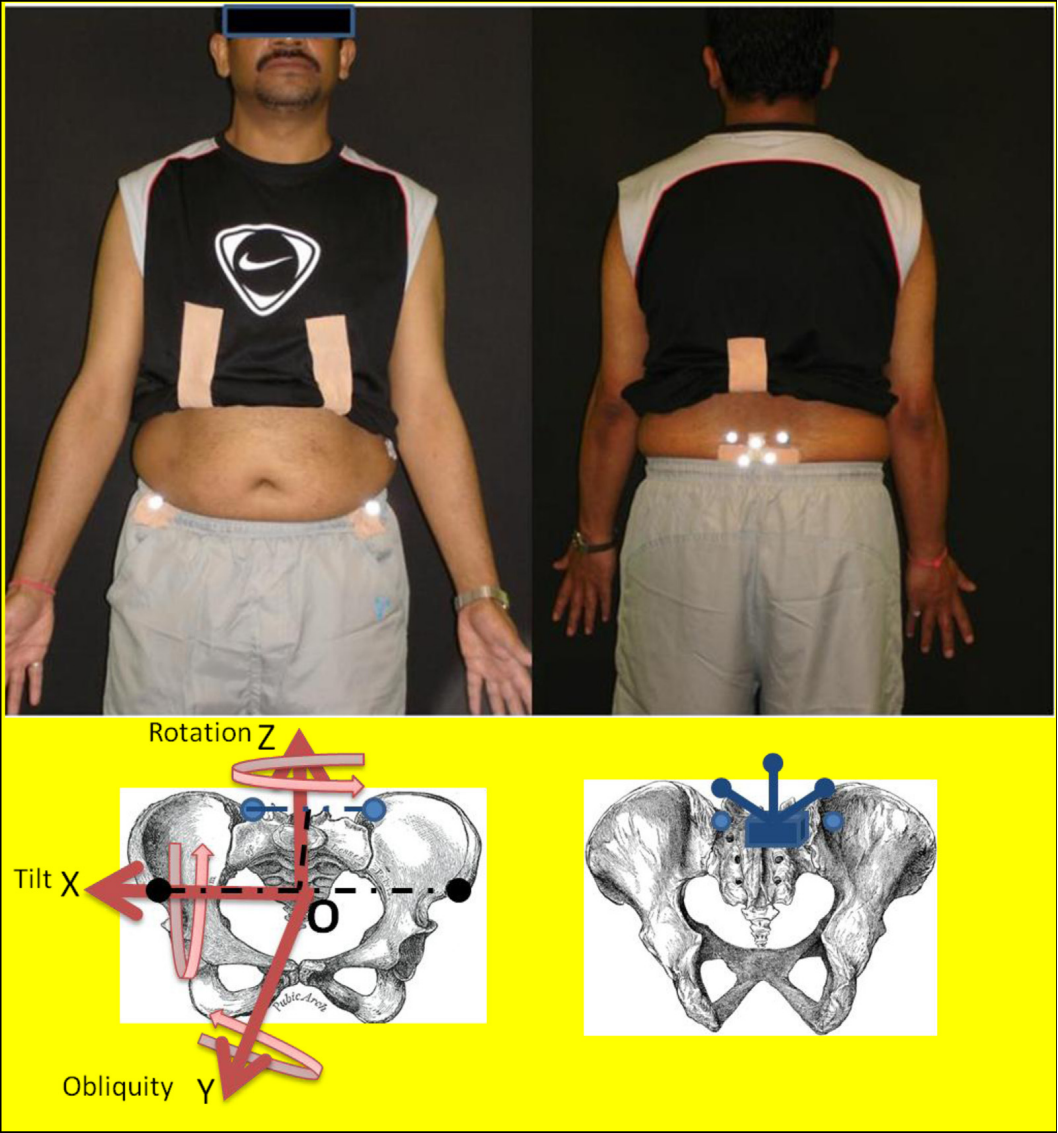
Shows the markers placed on boney landmarks of the pelvis. Top left picture shows the anterior view of a subject with two markers on the ASIS and top right picture shows the posterior view of two markers placed on the PSIS and the cluster of three markers attached to the sacrum. For the Traditional set four anatomical markers are used to track the motion (two black circles = left/right ASIS and two light blue circles = left/right PSIS are shown on the skeleton) while for the Cluster method, a separate cluster positioned on sacrum is used for tracking the pelvic movement which is shown by blue colour on the bottom left picture. Coordinate frame of the pelvis is in red. Pelvic tilt represents the movement of the pelvis around the *X* axis (flexion/extension), pelvic obliquity shows the movement of the pelvis around the *Y* axis (Abduction/adduction), and finally pelvic rotation stands for the movement of the pelvis around the *Z* axis. The origin of the segment is defined as the midpoint between two ASIS, *X* axis defined as a line parallel to the ASIS () and the *Y* axis is defined as a line connecting the midpoints of ASIS and PSIS (- - - - - -). The *Z* axis is orthogonal to other two axes. (For interpretation of the references to color in this figure legend, the reader is referred to the web version of this article.)

Over the past decade the understanding of pelvic kinematics during gait has increased despite a lack of clearly defined measurement standards. The most commonly used model in gait analysis is the kinematic model described by Kadaba et al. [1] and Davis et al. [2]. In the latter model, calculation of lower limb kinematics is based on the anterior superior iliac spines (ASIS) therefore occlusion of these markers for all or part of the trial will result in loss of some data. Occlusion of the ASIS could be as a result of soft tissue around the anterior abdomen (a common issue in overweight and obese subjects), arm movement, or activities that require high degrees of hip and trunk flexion, such as running, stair climbing or level walking [3]. One known modification to overcome ASIS occlusion is to introduce two technical markers to the pelvis positioned an equal distance laterally and posteriorly to the ASIS marker (often placed on the iliac crest) [4]. In order to use these technical markers, the ASIS marker positions can be expressed in relation to a technical coordinate system created using the technical markers in a static trial where the subject is stationary for couple of seconds with both anatomical and technical markers on the pelvis. However, having these technical markers on the lateral side of the waist does not guarantee reliable results, as again this is a site for fat deposition and substantial amount of fat and skin tissue may be present. There are no reports on how this method could be reliable for overweight and obese subjects. Generally, in the previous studies there has been no reporting on how to minimise the soft tissue artefact for overweight and obese subjects performing range of motion activities. Another previously used method involved a triad of markers directly placed on the posterior aspect of the pelvis. This was used to define directly the pelvic anatomical coordinate frame [5,6]. Pohl et al. [6] similarly used a rigid triad of markers to describe pelvic kinematics with the addition of two markers on the iliac crest, noting that this may not be the most reliable method to define the frontal plane of the pelvis [6]. This study proposed a potential solution to this problem which is the use of a cluster of three orthogonal markers attached to a rigid based as technical markers. This cluster is attached to the sacrum (Fig. 1) as this provides more accurate results than the ASIS and has less skin artefact [7]. Using the ‘calibrated anatomical system technique’ (CAST) [8,10] allows the position of ASIS defined relative to the Cluster in a static trial and then during dynamic trial the position of the ASIS is linked to the Cluster and thus affected by the same skin movement artefact that affects the Cluster [11]. The aim of this study is to compare the Cluster method with the Traditional method, which is the use of four surface markers on the right and left anterior superior iliac spine and left and right posterior superior iliac spine, in a population of healthy volunteers with varying body mass index (BMI).

## 2 Methodology

### 2.1 Participants

Thirty healthy subjects participated in this study (mean ± SD age and body mass index of 32.5 ± 12.3 years, and 26.39 ± 4.20 kg/m^2^, respectively). They were divided in three equal groups of normal, overweight, and obese according to their body mass index (BMI) (normal 19–24 kg/m^2^, overweight 24–28 kg/m^2^, and obese 28–35 kg/m^2^). None of the subjects had any history of lower back pain, surgery on the hip or lower limbs. They had no musculoskeletal injuries or disorders that affect walking ability. Written informed consent was obtained prior to participation. This study was approved by the Imperial College Research Ethics Committee (ICREC).

### 2.2 Data collection

An optical motion tracking system (VICON, Oxford, UK) consisting of nine high speed MX-13+ cameras was used at acquisition rate of 150 Hz. The same assessor carried out all data collection and analysis. Spherical reflective markers of 14 mm in diameter were applied concurrently (Fig. 1): (a) RASIS, LASIS, LPSIS, and RPSIS (Traditional); (b) a rigid cluster of three markers on sacrum (Cluster). In addition, three markers were attached to boney landmarks on the right and left foot to determine toe-off events. Markers location and segment definitions are described in Table 1.

Each subject was recorded in three sessions, one week apart. The subjects were asked to stand still while LASIS and RASIS were calibrated using the tip of the calibration wand (which is an L-frame used by VICON for the calibration of capturing volume) of known dimensions as proposed by Cappozzo et al. [9]. The wand's technical coordinate frame was then used to define the position of each ASIS with respect to the coordinate frame of the cluster. Following this, a static trial was conducted to allow the cameras to record the marker positions of the Traditional method; this includes the positions of the PSIS markers that are then defined with respect to the cluster for the Cluster method. Vicon Nexus 1.7.1 and Vicon BodyBuilder 3.6.1 were used to capture and process the data.

Each subject was asked to complete five trials in each session for eight different activities of daily living: (1) walking at self selected speed (walking), (2) standing up from standard sitting position, walk a distance of 2 m, turn and back to the chair and sitting down(Time up), (3) picking up a light box from the floor by bending their knees (Box), (4) sitting and standing from a backless chair (Sit-to-Stand), (5) reaching towards the toes without bending the knees (Toe), (6) squatting until they feel the seat (Squat), (7) ascending the stairs (Up-stairs), and (8) descending the stairs (Down-stairs).

### 2.3 Data analysis

The data for one stride (between two successive left- toe offs) of each trial were time normalised from 0 to 100% of the gait cycle and for activities involved the full range of motion of the pelvis such as Box, Toe and Sit-to-Stand, the data were normalised to 100% of the pelvis movement defined from 20 ms prior to start the task to 20 ms after finishing the task. The data were filtered using a 4th order low-pass Butterworth filter with cut off frequency of 6 Hz. In this study the Left side (left leg) were selected arbitrarily.

The pelvis angles were calculated using XYZ Cardan rotation sequence (tilt, obliquity, and rotation) which is the conventional sequence in many commercial gait analysis software packages (Vicon Clinical Manager: Oxford Metrics, UK) [12]. For each subject, standard deviations of the discrete parameters were calculated using key features that were consistently identifiable in both sets which were maximum pelvic tilt, maximum pelvic obliquity, and maximum pelvic rotation [10,13]. Intra-session variability was assessed for maximum pelvic tilt, pelvic obliquity and pelvic rotation by taking their averaged standard deviations (SD) over three sessions for all ADLs among five trials for each session (intra-session SD-variability). As the marker placement did not change between the trials in each session, the intra-variability is an indicator of repeatability of the subjects’ performance within each session. Inter-session variability was quantified by calculating the SD for the average of the five trials between the sessions. This illustrates the consistency of the subjects’ performance as well as the system's performance from one day to the other.

For each subject, coefficient of multiple correlation (CMC), was used to describe the repeatability of kinematic data using the waveform of each ADL for within (wCMC) and between (bCMC) sessions, with greater than 0.8 indicating high repeatability. Inter-protocol coefficient of multiple correlation (ipCMD) was used to evaluate the overall similarities between the waveforms of the two methods [14,15].

ANOVA for repeated measures was selected to obtain the kinematic differences between the two methods, activities of daily living, and body mass index.

## 3 Results

Intra-session and inter-session of mean standard deviation of maximum pelvic tilt for walking and some of the daily living activities that required full range of movement of the pelvis are summarised in Table 2 (results for the rest of the activities are available online).

For intra-session SD of normal subjects, there was no significant difference between the two methods for non-rotational planes (tilt *p* = 0.31 and obliquity *p* = 0.14) while for inter-session SD there was no significant difference between the two methods in all planes (tilt *p* = 0.23, obliquity *p* = 0.16, rotation *p* = 0.50). On average for overweight and obese subjects, the standard deviation of mean pelvic tilt using the Traditional was significantly higher than that of the Cluster method for both intra and inter-session (*p* < 0.05). The performance of each method during activities of daily living is also compared individually. Table 2 summarised the result obtained for normal, overweight and obese subjects during activities such as Box, Sit-to-Stand, Toe, Squat and walking (extra online material is provided for other activities). The results for overweight and obese subjects shows that the intra-session variability of the kinematic data using the Traditional method is significantly higher than that of the Cluster method in sagittal plane for activities that involves the full range of pelvic motion (*p* < 0.05).

Table 3 summarises the within-day, between day CMC results. The w and bCMC values obtained by two methods for each activity of daily living were compared between the three groups (detailed data are available online). The result shows that on average there are no significant differences between the repeatability of the kinematic waveforms between the two methods for normal subjects across all activities (tilt *p* = 0.21, obliquity *p* = 0.09, rotation *p* = 0.11). For activities that involve the full range of motion of pelvis in the sagittal plane, the b and wCMC values are significantly higher than those of the activities that involve a small movement of pelvis in sagittal plane (*p* < 0.05).

The inter-protocol CMC values are also summarised in Table 4. Higher values of ipCMC represent the similarity between the waveforms. As shown in Table 4, normal subjects have higher ipCMC values in comparison to the overweight and obese subjects in all planes.

## 4 Discussion

Establishing the repeatability of measuring three-dimensional angular kinematics of the pelvis during different daily living activities is critical if one wishes to distinguish the pathological changes from technical or experimental artefacts [16].

This study demonstrated that the pelvic kinematics in the sagittal plane during gait shows a high level of repeatability for both the Cluster and Traditional methods (Table 3). Comparing the bCMC from previous studies [15,17], both set of markers results were higher in all non-rotational values. As CMC is based on the ratio of error variance to true variance, therefore the low bCMC value of pelvic tilt in previous studies [13,15,17] may be related to a smaller range of motion of the pelvis during walking. In this study, activities of daily living such as Squat, Sit-to-Stand, Box, or Toe involved the full range of motion of the pelvis in the sagittal plane with little or no movement in the transverse and frontal planes. Therefore the CMC values obtained from kinematic waveform for such activities were higher due to the larger range of motion of the pelvis.

This study also compared the influence of BMI on repeatability of pelvic kinematics. The wCMC and bCMC values for overweight and obese subjects showed a significantly higher repeatability for the Cluster method than that of the Traditional method in all planes (Table 3 and online table). The moderate [18] results of bCMC for the Traditional method may indicate difficulty with occlusion of ASIS markers and soft tissue artefact during data collection for overweight and obese subjects.

Supplementary material related to this article found, in the online version, at http://dx.doi.org/10.1016/j.gaitpost.2013.05.019.



Standard deviation was also selected to quantify variability between marker sets for normal, overweight and obese subjects (Table 2). Inter-session variability was higher than the intra-session variability. This is due to the fact that intra-session variability is not impacted by marker placement differences while inter-session variability includes changes in the subject's walking pattern from day to day that are part of the natural variability of the subject as well as marker placement differences. The intra and inter session variability of the Cluster method is lower than that of the Traditional method especially for overweight and obese subjects. Higher variability in the Traditional method may arise from soft tissue artefact, marker occlusion during the data collection due to excess of soft tissue (for obese subjects); while introducing the technical frame and the concept of anatomical landmark calibration [9] in the Cluster method minimised the effect of soft tissue artefact. This fact can be explained further by comparing the performance of the two methods across activities that involves higher range of pelvic motion therefore more prone to soft tissue artefact. This showed that for activities such as Squat, Box, Sit-to-Stand and Toe the intra and inter session variability was significantly (*p* < 0.05) higher for the Traditional method than the Cluster method for overweight and obese subjects in the sagittal plane and there were no significant differences between the two methods for such activities in normal subjects (*p* = 0.28). As the soft tissue artefact is not consistent from one trial to the next, the high variability of the Traditional method in such activities may be as a result of such errors as well as movement of the markers independently relative to each other. For activities that require less movement of the pelvis such as walking, Up-stairs and Down-stairs there were no significant differences between the two methods for intra and inter variability for different BMI groups (*p* = 0.48, *p* = 0.09). For activities that involved speed (Time up), significant differences (*p* < 0.05) were found between the two methods in the sagittal plane for obese and overweight subjects (intra and inter-session). Details of these results are available on line.

In addition to standard deviation, the similarity between the two marker sets was reported using ipCMC (Table 4). The low ipCMC values for overweight and obese groups indicate the poor similarity between the two methods while for normal subjects there is a good similarity. To determine whether the cluster mounted on the sacrum does minimise the effect of the soft tissue artefact, we can compare the result of this study with Bull and McGregor [7] in which they demonstrated that it is possible to accurately measure the motion of the lumbo-sacral spine using a sensor attached to the sacrum and provide useful and important information on the motion of the body segments during rowing with average error of ±1.0°.

## 5 Conclusion

Both marker sets generally showed high repeatability for all three subject groups, while for overweight and obese subjects the Cluster method showed significantly better repeatability than that of the Traditional method. Both methods were comparable in the measurement of gait with the Traditional method demonstrating high level of repeatability. This is not surprising as this is what the Traditional method was originally intended to measure. The Cluster method overcomes a number of theoretical and experimental limitations such as minimising the effect of movement of markers relative to each other as well as to the underlying bone, fewer cameras are required to track the cluster with implication for cost and laboratory set up procedures. Also less time is needed for post processing the data as there is no marker occlusion in the dynamic trials therefore no further programming is needed to fill the gaps in dynamic trials.

This study provides evidence that a new technical marker set is superior for three-dimensional data collection of overweight and obese subjects, and when the ASIS markers are occluded for all or part of the trial particularly during a range of activity of daily living. The accuracy of both marker sets to follow the underlying bone movement was not determined in this study and warrants further investigation. Notwithstanding these limitations, a repeatable measure of pelvic motion has been tested in this study.

## Conflict of interest statement

The authors had no conflict of interest when performing the study or when preparing the manuscript.

## Figures and Tables

**Table 1:** Definitions of boney landmarks for the Cluster and Traditional sets. These anatomical sets were used to define the segment coordinate frame.

Anatomical sets	Description	Identification
*Cluster method*		
L/R ASIS	Most prominent point of left and right ASIS	Pointer
L/R PSIS	Most prominent point of left and right PSIS	Marker
Technical set for pelvis		
Marker cluster	Rigid cluster of 3 markers placed sacrum	Marker

*Traditional method*		
L/R ASIS	Most prominent point of left and right ASIS	Marker
L/R PSIS	Most prominent point of left and right PSIS	Marker

*Definition of segment coordinate frame*		
Pelvis		
*O*	Midpoint between ASISs	
*X*	Parallel to the line connecting ASISs, positive to the right	
*Z*	Orthogonal to the plane defined by ASISs and PSISs, positive superiorly	
*Y*	Orthogonal to other two axes, positive anteriorly	

L/R represents left/right.

**Table 2:** Intra-session and inter-session means of standard deviation of maximum pelvic tilt, obliquity and rotation for activities of daily living that involves the full range of the motion of pelvis and walking.

(*n* = 30)		Cluster method (±SD)			Traditional method (±SD)		
		Tilt	Obliquity	Rotation	Tilt	Obliquity	Rotation
*Intra-session*							
BOX	Normal	5.51 (2.54)	2.65 (0.87)	2.54 (1.83)	5.57 (3.98)	2.71 (1.20)	1.76 (0.79)
	Overweight	2.25 (2.93)*	**2.76** (**1.52)**	1.73 (0.62)	**5.88** (**3.86)**	0.87 (0.54)*	1.23 (0.59)
	Obese	2.60 (1.48)*	2.01 (0.95)	**1.99** (**1.35)**	**6.16** (**4.22)**	1.08 (0.49)	0.83 (0.44)*
Squat	Normal	6.45 (1.78)	2.83 (0.92)	2.42 (1.24)	5.50 (1.59)	2.13 (0.71)	1.89 (1.00)
	Overweight	4.37 (2.85)*	2.60 (1.46)	1.91 (1.26)	**7.77** (**4.76)**	1.25 (0.47)	1.66 (0.93)
	Obese	2.96 (1.54)*	**2.25** (**1.46)**	2.40 (1.52)	**4.69** (**2.39)**	0.80 (0.41)*	1.33 (0.62)
STS	Normal	5.25 (2.84)	2.55 (0.99)	4.06 (1.71)	5.05 (1.92)	2.46 (0.66)	5.45 (7.01)
	Overweight	3.16 (3.16)*	2.56 (1.85)	1.95 (1.18)	**5.42** (**4.18)**	1.39 (1.34)	1.25 (0.58)
	Obese	1.46 (1.08)*	1.62 (1.07)	2.11 (1.21)	**3.35** (**2.95)**	1.08 (1.32)	1.26 (1.05)
Toe	Normal	4.85 (1.95)	2.51 (0.92)	3.87 (2.70)	6.77 (3.72)	3.89 (1.72)	2.96 (1.90)
	Overweight	3.90 (2.75)*	2.80 (2.01)	1.91 (0.75)	**5.73 (3.63)**	2.49 (2.03)	1.57 (0.85)
	Obese	2.95 (2.32)*	2.19 (1.13)	2.04 (1.19)	**5.31 (4.24)**	1.35 (0.94)	1.20 (0.33)
Walking	Normal	3.15 (2.10)	2.98 (1.31)	2.22 (0.90)	2.99 (2.09)	2.13 (0.57)	1.69 (0.84)
	Overweight	3.57 (1.49)	2.87 (1.02)	2.36 (1.07)	3.80 (1.69)	2.30 (0.88)	1.54 (0.58)
	Obese	2.20 (1.50)	1.97 (0.57)	2.27 (1.84)	1.47 (0.97)	2.09 (0.64)	1.07 (0.57)

*Inter-session*							
BOX	Normal	6.91 (3.88)*	3.04 (1.75)	4.32 (1.45)	**7.50 (6.32)**	3.15 (1.13)	5.08 (2.43)
	Overweight	5.82 (3.44)*	2.94 (0.76)	4.37 (1.41)	**8.35 (2.26)**	3.38 (1.73)	3.90 (1.91)
	Obese	4.12 (3.43)*	1.89 (0.35)	4.21 (0.94)	**8.43 (3.96)**	2.93 (1.92)	3.94 (1.93)
Squat	Normal	4.04 (2.81)	2.74 (4.22)	3.81 (0.85)	3.33 (2.07)	2.29 (3.57)	4.21 (1.61)
	Overweight	3.93 (1.37)*	2.84 (1.26)	4.21 (1.82)	**5.98 (1.45)**	2.52 (1.05)	3.59 (1.19)
	Obese	4.47 (1.99)*	1.85 (0.87)	2.84 (1.00)	**6.17 (2.94)**	1.94 (0.75)	3.30 (1.42)
STS	Normal	3.68 (1.82)*	4.47 (4.42)	9.66 (2.20)	**5.38 (2.77)**	4.79 (3.94)	9.71 (2.05)
	Overweight	4.91 (1.24)*	2.72 (1.08)*	3.45 (1.30)	**6.05 (2.35)**	**4.67 (4.95)**	3.20 (1.31)
	Obese	5.81 (2.91)*	2.15 (0.72)	3.69 (1.42)	**9.11 (9.18)**	4.22 (5.53)	4.18 (3.55)
Toe	Normal	4.86 (2.88)	2.76 (2.49)	2.84 (1.56)	5.49 (3.44)	3.61 (3.41)	1.57 (0.93)
	Overweight	4.69 (1.71)*	2.76 (1.02)	3.15 (1.75)	**7.40 (3.90)**	3.87 (2.51)	2.67 (1.16)
	Obese	4.47 (1.53)*	2.05 (0.64)	2.08 (1.15)	**7.70 (3.83)**	3.12 (1.82)	2.10 (0.80)
Walking	Normal	4.91 (2.20)	2.63 (1.79)	5.70 (3.16)	4.30 (1.97)	2.81 (0.38)	5.04 (1.36)
	Overweight	2.83 (2.20)	2.41 (1.19)	5.49 (2.85)	2.28 (1.94)	2.96 (0.71)	5.18 (2.02)
	Obese	3.89 (1.07)	1.98 (1.23)	4.66 (1.17)	3.60 (1.00)	0.71 (0.51)	4.63 (0.59)

STS = Sit-to-Stand.

* Highlights statistically significant differences between two sets (*p* < 0.05) with bold value higher.

**Table 3:** Coefficient of multiple correlation averages (CMC) and its standard deviation for within, between day (w, b).

(*n* = 30)		Cluster method (±SD)			Traditional method (±SD)		
		Tilt	Obliquity	Rotation	Tilt	Obliquity	Rotation
*Within-day CMC*							
BOX	Normal	0.92 (0.05)	0.70 (0.18)	0.87 (0.11)	0.93 (0.06)	0.88 (0.11)	0.84 (0.12)
	Overweight	0.98 (0.02)	0.96 (0.03)	0.95 (0.02)	0.97 (0.02)	0.92 (0.07)	0.93 (0.04)
	Obese	0.98 (0.02)	0.96 (0.04)	0.96 (0.02)	0.98 (0.02)	0.92 (0.06)	0.94 (0.04)
Squat	Normal	0.98 (0.01)	0.97 (0.03)	0.97 (0.01)	0.97 (0.02)	0.94 (0.05)	0.95 (0.02)
	Overweight	**0.99 (0.01)**	0.97 (0.03)	0.96 (0.03)	0.97 (0.04)*	0.91 (0.15)	0.93 (0.05)
	Obese	**0.99 (0.01)**	0.98 (0.03)	0.98 (0.01)	0.97 (0.04)*	0.94 (0.03)	0.92 (0.08)
STS	Normal	0.99 (0.01)	0.99 (0.01)	0.96 (0.03)	0.99 (0.01)	0.96 (0.04)	0.93 (0.12)
	Overweight	0.99 (0.01)	0.98 (0.01)	0.96 (0.03)	0.98 (0.02)	0.91 (0.09)	0.90 (0.14)
	Obese	**0.99** (**0.01)**	0.98 (0.01)	0.97 (0.02)	0.97 (0.03)*	0.91 (0.12)	0.92 (0.06)
Toe	Normal	0.99 (0.01)	0.97 (0.03)	0.96 (0.03)	0.99 (0.01)	0.94 (0.03)	0.95 (0.04)
	Overweight	1.00 (0.00)	0.97 (0.02)	0.96 (0.02)	0.99 (0.01)	0.96 (0.03)	0.96 (0.03)
	Obese	**0.99** (**0.01)**	0.97 (0.03)	0.98 (0.02)	0.98 (0.03)*	0.93 (0.08)	0.96 (0.03)
Walking	Normal	**0.93** (**0.04)**	0.98 (0.01)	0.96 (0.02)	0.89 (0.06)*	0.98 (0.01)	0.96 (0.02)
	Overweight	**0.92** (**0.04)**	0.99 (0.01)	0.97 (0.02)	0.86 (0.06)*	0.99 (0.01)	0.96 (0.02)
	Obese	**0.96** (**0.02)**	0.99 (0.01)	0.97 (0.01)	0.91 (0.05)*	0.98 (0.02)	0.96 (0.02)

*Between-day CMC*							
BOX	Normal	0.92 (0.05)	0.86 (0.10)	0.87 (0.11)	0.93 (0.06)	0.88 (0.11)	0.84 (0.12)
	Overweight	**0.93 (0.10)**	**0.91 (0.06)**	0.87 (0.12)	0.90 (0.11)*	0.72 (0.24*	0.90 (0.07)
	Obese	**0.99 (0.01)**	**0.91 (0.07)**	0.94 (0.05)	0.94 (0.04)*	0.64 (0.25*	0.83 (0.14)
Squat	Normal	0.93 (0.10)	0.78 (0.28)	0.82 (0.15)	0.95 (0.04)	0.65 (0.29)	0.79 (0.15)
	Overweight	**0.95 (0.09)**	**0.85 (0.12)**	0.81 (0.11)	0.92 (0.11)*	0.68 (0.22*	0.80 (0.15)
	Obese	**0.98 (0.02)**	**0.90(0.08)**	0.79(0.18)	0.93(0.06)*	0.65(0.28*	0.85(0.09)
STS	Normal	0.97(0.02)	0.73(0.16)	0.77(0.25)	0.98(0.01)	0.80(0.19)	0.77(0.26)
	Overweight	0.97(0.02)	**0.91(0.07)**	0.87(0.14)	0.96 (0.03)	0.78 (0.16*	0.86 (0.12)
	Obese	0.97 (0.02)	**0.94 (0.08)**	0.90 (0.12)	0.98 (0.02)	0.86 (0.13*	0.89 (0.08)
Toe	Normal	0.98 (0.02)	0.79 (0.09)	0.81 (0.09)	0.97 (0.03)	0.67 (0.28)	0.82 (0.11)
	Overweight	0.98 (0.04)	**0.82 (0.11)**	0.77 (0.21)	0.98 (0.02)	0.65 (0.23*	0.79 (0.15)
	Obese	**0.99 (0.02)**	**0.87 (0.11)**	0.84 (0.10)	0.96 (0.04)*	0.67 (0.22*	0.75 (0.24)
Walking	Normal	0.81 (0.12)	0.98 (0.02)	0.97 (0.04)	0.74 (0.23)	0.89 (0.12)	0.97 (0.02)
	Overweight	0.75 (0.19)	**0.98 (0.03)**	0.96 (0.03)	0.76 (0.15)	0.89 (0.11*	0.94 (0.04)
	Obese	0.85 (0.12)	0.90 (0.05)	0.95 (0.02)	0.87 (0.12)	0.99 (0.01)	0.97 (0.03)

* Highlights statistically significant differences between two sets (*p* < 0.05) with bold value higher.

**Table 4:** Inter-protocol coefficient of multiple correlations for walking and activities of daily living involving full range of motion.

Inter-protocol CMC						
(*n* = 30)		Box	Squat	STS	Toe	Walking
Pelvic tilt	Normal	0.68 (0.26)	0.70 (0.28)	0.86 (0.11)	0.86 (0.21)	0.55 (0.44)
	Overweight	0.56 (0.41)	0.65 (0.36)	0.65 (0.29)	0.79 (0.27)	−0.04 (0.36)
	Obese	0.54 (0.32)	0.49 (0.33)	0.46 (0.25)	0.63 (0.31)	−0.08 (0.34)
Pelvic obliquity	Normal	0.19 (0.51)	0.19 (0.53)	0.19 (0.48)	0.02 (0.48)	0.59 (0.33)
	Overweight	0.05 (0.45)	0.01 (0.47)	0.03 (0.35)	0.00 (0.38)	0.46 (0.30)
	Obese	−0.04 (0.34)	−0.10 (0.31)	0.18 (0.30)	−0.11 (0.39)	0.37 (0.37)
Pelvic rotation	Normal	0.46 (0.34)	0.36 (0.36)	0.26 (0.45)	0.24 (0.44)	0.78 (0.13)
	Overweight	0.35 (0.38)	0.31 (0.43)	0.31 (0.38)	0.16 (0.47)	0.69 (0.26)
	Obese	0.20 (0.45)	0.06 (0.47)	0.10 (0.43)	−0.10 (0.48)	0.55 (0.26)
