# Supplementary material for: An alternative technical marker set for the pelvis is more repeatable than the standard pelvic marker set
Source: Gait Posture. 2013 Sep;38(4):1032–7. doi: 10.1016/j.gaitpost.2013.05.019 (PMC3989066; doi:10.1016/j.gaitpost.2013.05.019)
Supplement: Supplementary file 1 [file mmc1.docx]

| **(n=10)** | | **BOX(sd)** | | | | **Squat(sd)** | | | **Stairs down** | | | **Stairs-up** | | | **STS** | | | **Time-up** | | | **Toe** | | | **Walking** | | |
| --- | --- | --- | --- | --- | --- | --- | --- | --- | --- | --- | --- | --- | --- | --- | --- | --- | --- | --- | --- | --- | --- | --- | --- | --- | --- | --- |
|  |  | **CM** | | **TM** | | **CM** | **TM** | | **CM** | **TM** | | **CM** | **TM** | | **CM** | **TM** | | **CM** | | **TM** | **CM** | | **TM** | **CM** | | **TM** |
| **Within-day CMC (±SD)** | | | | | | | | | | | | | | | | | | | | | | | | | | |
| **Normal** | **T** | 0.922  (0.050) | | 0.928  (0.059) | | 0.981  (0.011) | 0.973  (0.019) | | **0.893**  **(0.082)** | 0.800  (0.253) | | 0.992  (0.067) | 0.826  (0.156) | | 0.990  (0.006) | 0.993  (0.005) | | 0.981  (0.009) | | 0.963  (0.028) | 0.995  (0.005) | | 0.990  (0.01) | **0.929**  **(0.043)** | | 0.889  (0.064) |
|  | **O** | 0.701  (0.184) | | 0.879  (0.106) | | 0.974  (0.026) | 0.942  (0.046) | | 0.973  (0.024) | 0.965  (0.039) | | 0.967  (0.033) | 0.955  (0.054) | | 0.987  (0.011) | 0.958  (0.040) | | 0.982  (0.02) | | 0.983  (0.014) | 0.967  (0.033) | | 0.937  (0.034) | 0.984  (0.015) | | 0.984  (0.015) |
|  | **R** | 0.870  (0.107) | | 0.838  (0.122) | | 0.966  (0.015) | 0.954  (0.025) | | **0.920**  **(0.058)** | 0.888  (0.171) | | 0.998  (0.002) | 0.989  (0.019) | | 0.961  (0.031) | 0.926  (0.119) | | 0.991  (0.014) | | 0.994  (0.014) | 0.958  (0.031) | | 0.951  (0.044) | 0.957  (0.019) | | 0.960  (0.024) |
| **Overweight** | **T** | 0.978  (0.024) | | 0.973  (0.021) | | **0.993**  **(0.006)** | 0.975  (0.038) | | **0.955**  **(0.017)** | 0.872  (0.100) | | **0.959**  **(0.022)** | 0.881  (0.103) | | 0.991  (0.007) | 0.981  (0.020) | | 0.990  (0.006) | | 0.980  (0.014) | 0.996  (0.002) | | 0.988  (0.015) | **0.919**  **(0.044)** | | 0.860  (0.058) |
|  | **O** | 0.963  (0.030) | | 0.925  (0.071) | | 0.968  (0.026) | 0.906  (0.147) | | 0.981  (0.018) | 0.970  (0.030) | | 0.975  (0.020) | 0.981  (0.014) | | 0.979  (0.012) | 0.911  (0.088) | | 0.991  (0.006) | | 0.996  (0.002) | 0.970  (0.024) | | 0.962  (0.035) | 0.988  (0.005) | | 0.990  (0.006) |
|  | **R** | 0.953  (0.024) | | 0.925  (0.036) | | 0.959  (0.033) | 0.927  (0.047) | | 0.963  (0.026) | 0.938  (0.045) | | 0.999  (0.001) | 0.999  (0.001) | | 0.958  (0.030) | 0.903  (0.139) | | 0.994  (0.017) | | 0.999  (0.001) | 0.963  (0.018) | | 0.961  (0.028) | 0.956  (0.024) | | 0.961  (0.023) |
| **Obese** | **T** | 0.984  (0.022) | | 0.978  (0.017) | | **0.993**  **(0.005)** | 0.972  (0.038) | | **0.946**  **(0.024)** | 0.845  (0.080) | | **0.947**  **(0.032)** | 0.851  (0.099) | | **0.993**  **(0.005)** | 0.969  (0.035) | | 0.999  (0.005) | | 0.921  (0.087) | **0.993**  **(0.005)** | | 0.982  (0.032) | **0.962**  **(0.018)** | | 0.909  (0.055) |
|  | **O** | 0.963  (0.037) | | 0.921  (0.059) | | 0.976  (0.027) | 0.945  (0.026) | | 0.972  (0.039) | 0.948  (0.055) | | 0.984  (0.008) | 0.976  (0.031) | | 0.985  (0.010) | 0.912  (0.119) | | 0.999  (0.004) | | 0.922  (0.016) | 0.972  (0.031) | | 0.932  (0.078) | 0.998  (0.008) | | 0.979  (0.021) |
|  | **R** | 0.960  (0.024) | | 0.942  (0.037) | | 0.983  (0.013) | 0.920  (0.080) | | 0.951  (0.030) | 0.948  (0.018) | | 0.999  (0.001) | 0.992  (0.022) | | 0.975  (0.019) | 0.917  (0.061) | | 0.998  (0.002) | | 0.965  (0.002) | 0.978  (0.023) | | 0.957  (0.033) | 0.975  (0.013) | | 0.959  (0.018) |
| **Between-day CMC (±SD)** | | | | | | | | | | | | | | | | | | | | | | | | | | |
| **Normal** | **T** | 0.923  (0.050) | | 0.928  (0.059) | | 0.928  (0.096) | 0.954  (0.045) | | 0.710  (0.130) | 0.679  (0.281) | | 0.820  (0.180) | 0.737  (0.171) | | 0.970  (0.021) | 0.983  (0.015) | | 0.963  (0.032) | | 0.959  (0.038) | 0.978  (0.022) | | 0.975  (0.026) | 0.812  (0.121) | | 0.739  (0.227) |
|  | **O** | 0.860  (0.101) | | 0.879  (0.106) | | 0.785  (0.280) | 0.648  (0.291) | | 0.858  (0.155) | 0.967  (0.033) | | 0.933  (0.082) | 0.975  (0.018) | | 0.730  (0.161) | 0.797  (0.194) | | 0.966  (0.083) | | 0.979  (0.038) | 0.789  (0.094) | | 0.667  (0.283) | 0.985  (0.016) | | 0.893  (0.121) |
|  | **R** | 0.870  (0.107) | | 0.838  (0.123) | | 0.816  (0.149) | 0.792  (0.150) | | 0.912  (0.116) | 0.927  (0.089) | | **0.901**  **(0.065)** | 0.787  (0.288) | | 0.770  (0.251) | 0.766  (0.260) | | 0.945  (0.113) | | 0.948  (0.108) | 0.813  (0.093) | | 0.822  (0.114) | 0.967  (0.036) | | 0.967  (0.024) |
| **Overweight** | **T** | **0.934**  **(0.103)** | | 0.896  (0.106) | | **0.946**  **(0.086)** | 0.922  (0.107) | | **0.808**  **(0.208)** | 0.692  (0.169) | | **0.762**  **(0.292)** | 0.676  (0.234) | | 0.972  (0.023) | 0.962  (0.030) | | 0.972  (0.025) | | 0.970  (0.031) | 0.976  (0.040) | | 0.979  (0.020) | 0.755  (0.192) | | 0.758  (0.147) |
|  | **O** | **0.907**  **(0.062)** | | 0.725  (0.240) | | **0.846**  **(0.117)** | 0.680  (0.224) | | 0.937  (0.071) | 0.977  (0.018) | | 0.932  (0.057) | 0.983  (0.012) | | **0.912**  **(0.074)** | 0.777  (0.159) | | 0.988  (0.011) | | 0.993  (0.010) | **0.818**  **(0.110)** | | 0.650  (0.226) | **0.979**  **(0.028)** | | 0.889  (0.110) |
|  | **R** | 0.868  (0.119) | | 0.902  (0.070) | | 0.811  (0.111) | 0.805  (0.153) | | 0.942  (0.060) | 0.957  (0.049) | | 0.869  (0.094) | 0.897  (0.069) | | 0.874  (0.138) | 0.863  (0.121) | | 0.999  (0.002) | | 0.999  (0.001) | 0.774  (0.213) | | 0.794  (0.154) | 0.965  (0.029) | | 0.941  (0.036) |
| **Obese** | **T** | **0.986**  **(0.011)** | | 0.941  (0.042) | | **0.979**  **(0.016)** | 0.930  (0.060) | | 0.917  (0.053) | 0.698  (0.206) | | 0.912  (0.054) | 0.702  (0.237) | | 0.968  (0.024) | 0.980  (0.024) | | 0.942  (0.083) | | 0.970  (0.031) | **0.986**  **(0.018)** | | 0.961  (0.040) | 0.854  (0.117) | | 0.874  (0.117) |
|  | **O** | **0.913**  **(0.068)** | | 0.641  (0.255) | | **0.899**  **(0.077)** | 0.646  (0.276) | | 0.920  (0.085) | 0.985  (0.010) | | 0.938  (0.077) | 0.980  (0.034) | | **0.939**  **(0.079)** | 0.860  (0.129) | | 0.986  (0.013) | | 0.994  (0.003) | **0.868**  **(0.109)** | | 0.673  (0.219) | 0.903  (0.055) | | 0.986  (0.013) |
|  | **R** | 0.940  (0.050) | | 0.829  (0.136) | | 0.789  (0.178) | 0.852  (0.095) | | 0.937  (0.065) | 0.925  (0.082) | | 0.917  (0.100) | 0.905  (0.080) | | 0.904  (0.118) | 0.892  (0.084) | | 0.999  (0.001) | | 0.999  (0.001) | 0.838  (0.104) | | 0.746  (0.239) | 0.947  (0.025) | | 0.969  (0.030) |
| **Intra-session Variability (±SD)** | | | | | | | | | | | | | | | | | | | | | | | | | | |
| **Normal** | **T** | 5.515  (2.542) | | 5.572  (3.978) | | 6.446  (1.780) | 5.497  (1.585) | | 5.642  (2.105) | 4.719  (1.694) | | 3.939  (1.677) | 3.817  (3.760) | | 5.249  (2.840) | 5.055  (1.917) | | 1.852  (4.133) | | 2.076  (8.418) | 4.847  (1.947) | | 6.769  (3.719) | 3.153  (2.103) | | 2.995  (2.091) |
|  | **O** | 2.646  (0.872) | | 2.709  (1.196) | | 2.831  (0.921) | 2.126  (0.709) | | 4.031  (0.840) | 3.546  (0.505) | | 7.094  (5.000) | 9.338  (7.851) | | 2.551  (0.988) | 2.459  (0.661) | | 1.539  (5.378) | | 1.532  (4.711) | 2.513  (0.922) | | 3.894  (1.724) | 2.977  (1.309) | | 2.134  (0.570) |
|  | **R** | 2.545  (1.831) | | 1.763  (0.790) | | 2.416  (1.238) | 1.891  (1.002) | | 2.414  (1.316) | 1.931  (1.187) | | 3.454  (1.682) | 3.970  (2.149) | | 4.057  (1.712) | 5.446  (7.010) | | 5.019  (2.157) | | 7.648  (2.287) | 3.872  (2.702) | | 2.958  (1.900) | 2.223  (0.897) | | 1.689  (0.841) |
| **Overweight** | **T** | 2.252  (2.927) | | **5.881**  **(3.857)** | | 4.372  (2.846) | **7.769**  **(4.759)** | | 3.087  (0.934) | 4.974  (1.777) | | 3.898  (0.537) | 4.176  (2.071) | | 3.160  (3.157) | **5.424**  **(4.183)** | | 7.429  (3.253) | | 8.586  (3.760) | 3.901  (2.747) | | **5.728**  **(3.633)** | 3.570  (1.495) | | 3.804  (1.695) |
|  | **O** | **2.756**  **(1.523)** | | 0.869  (0.541) | | 2.601  (1.464) | 1.246  (0.475) | | 3.532  (1.881) | 4.019  (2.096) | | 6.349  (2.024) | 5.431  (2.998) | | 2.559  (1.854) | 1.391  (1.337) | | 8.868  (3.650) | | **11.175**  **(5.624)** | 2.797  (2.014) | | 2.493  (2.027) | 2.870  (1.022) | | 2.298  (0.884) |
|  | **R** | 1.731  (0.620) | | 1.234  (0.587) | | 1.914  (1.265) | 1.662  (0.926) | | 3.409  (2.895) | 2.684  (3.836) | | 5.803  (3.390) | 5.221  (9.355) | | 1.955  (1.177) | 1.253  (0.577) | | 1.677  (8.533) | | 1.253  (6.273) | 1.914  (0.750) | | 1.566  (0.851) | 2.359  (1.077) | | 1.543  (0.580) |
| **Obese** | **T** | 2.597  (1.478) | | **6.159**  **(4.223)** | | 2.964  (1.539) | **4.686**  **(2.389)** | | 3.595  (1.164) | **6.184**  **(5.720)** | | 4.707  (2.639) | 4.787  (3.466) | | 1.459  (1.076) | **3.355**  **(2.950)** | | 4.765  (1.754) | | **7.511**  **(3.028)** | 2.947  (2.318) | | **5.308**  **(4.237)** | 2.200  (1.505) | | 1.471  (0.974) |
|  | **O** | 2.005  (0.954) | | 1.083  (0.486) | | **2.253**  **(1.464)** | 0.801  (0.415) | | 4.869  (4.433) | 6.399  (5.788) | | 2.442  (1.388) | 1.707  (1.425) | | 1.621  (1.067) | 1.078  (1.323) | | 3.940  (2.278) | | 3.308  (1.563) | 2.194  (1.133) | | 1.352  (0.937) | 1.968  (0.570) | | 2.086  (0.644) |
|  | **R** | **1.994**  **(1.351)** | | 0.832  (0.440) | | 2.405  (1.516) | 1.335  (0.616) | | 1.822  (5.421) | 1.973  (7.334) | | 1.400  (5.606) | 1.969  (6.832) | | 2.115  (1.214) | 1.256  (1.055) | | 1.472  (7.352) | | 2.515  (3.073) | 2.037  (1.191) | | 1.198  (0.333) | 2.266  (1.838) | | 1.067  (0.575) |
| **Inter-session Variability (±SD)** | | | | | | | | | | | | | | | | | | | | | | | | | | |
| **Normal** | **T** | 6.911  (3.884) | | **7.502**  **(6.323)** | | 4.042  (2.813) | 3.326  (2.075) | | 4.418  (2.073) | 3.299  (2.239) | | 5.457  (2.010) | 6.005  (9.584) | | 3.685  (1.824) | **5.379**  **(2.768)** | | 5.511  (1.812) | | 4.839  (3.719) | 4.856  (2.884) | | 5.493  (3.444) | 4.909  (2.204) | | 4.305  (1.974) |
|  | **O** | 3.043  (1.748) | | 3.146  (1.135) | | 2.744  (4.224) | 2.291  (3.567) | | 2.347  (2.313) | 1.388  (0.724) | | 2.947  (2.087) | 2.620  (1.567) | | 4.466  (4.423) | 4.793  (3.941) | | 5.623  (2.929) | | 4.806  (3.347) | 2.764  (2.491) | | 3.608  (3.407) | 2.627  (1.789) | | 2.811  (0.384) |
|  | **R** | 4.324  (1.454) | | 5.078  (2.431) | | 3.812  (0.852) | 4.206  (1.608) | | 9.419  (4.159) | 9.019  (3.841) | | 4.670  (5.168) | 5.476  (6.754) | | 9.663  (2.203) | 9.715  (2.047) | | 4.878  (7.866) | | 5.157  (7.224) | 2.839  (1.559) | | 1.574  (0.933) | 5.702  (3.158) | | 5.036  (1.364) |
| **Overweight** | **T** | 5.816  (3.437) | | **8.348**  **(2.263)** | | 3.932  (1.375) | **5.983**  **(1.452)** | | 3.636  (1.709) | 2.468  (2.045) | | 4.820  (2.353) | 5.582  (2.929) | | 4.912  (1.242) | **6.055**  **(2.350)** | | 2.961  (3.215) | | **5.575**  **(3.542)** | 4.692  (1.707) | | **7.397**  **(3.903)** | 2.834  (2.204) | | 2.277  (1.936) |
|  | **O** | 2.938  (0.765) | | 3.378  (1.733) | | 2.843  (1.265) | 2.523  (1.053) | | 2.126  (1.254) | 1.131  (0.589) | | 3.191  (1.745) | 1.653  (0.944) | | 2.717  (1.082) | **4.670**  **(4.949)** | | 2.961  (3.201) | | **5.719**  **(3.577)** | 2.764  (1.024) | | 3.870  (2.512) | 2.407  (1.189) | | 2.956  (0.708) |
|  | **R** | 4.368  (1.415) | | 3.899  (1.912) | | 4.213  (1.824) | 3.589  (1.191) | | 8.820  (4.424) | 9.483  (6.259) | | 5.696  (5.579) | 4.052  (2.729) | | 3.450  (1.301) | 3.196  (1.306) | | 5.523  (7.872) | | 7.757  (14.437) | 3.147  (1.749) | | 2.667  (1.165) | 5.493  (2.851) | | 5.179  (2.019) |
| **Obese** | **T** | 4.122  (3.428) | | **8.427**  **(3.959)** | | 4.475  (1.993) | **6.166**  **(2.941)** | | 1.686  (1.293) | **4.993**  **(3.442)** | | 3.195  (1.003) | 3.810  (1.425) | | 5.811  (2.910) | **9.113**  **(9.178)** | | 6.309  (1.996) | | **13.786**  **(12.943)** | 4.472  (1.532) | | **7.703**  **(3.831)** | 3.891  (1.072) | | 3.599  (1.000) |
|  | **O** | 1.892  (0.350) | | 2.926  (1.920) | | 1.848  (0.868) | 1.943  (0.755) | | 3.046  (2.613) | 1.174  (0.263) | | 4.020  (1.359) | 6.488  (3.791) | | 2.148  (0.718) | 4.224  (5.532) | | 7.143  (1.759) | | 8.175  (3.677) | 2.046  (0.641) | | 3.117  (1.825) | 1.976  (1.233) | | 0.712  (0.511) |
|  | **R** | 4.207  (0.941) | | 3.944  (1.926) | | 2.839  (1.005) | 3.3020  (1.418) | | 3.523  (1.735) | 3.076  (2.071) | | 2.308  (4.499) | 3.480  (2.077) | | 3.689  (1.418) | 4.182  (3.554) | | 5.095  (3.587) | | 4.704  (4.161) | 2.083  (1.150) | | 2.103  (0.802) | 4.656  (1.166) | | 4.635  (0.594) |
| **Inter- protocol CMC** | | | | | | | | | | | | | | | | | | | | | | | | | | |
|  | | |  | | **Box** | | | **Squat** | | | **STS** | | | **Toe** | | | **Walking** | | **Stair up** | | | **Stair down** | | | **Time up** | |
| Pelvic tilt | | | Normal | | 0.681  (0.264) | | | 0.696  (0.278) | | | 0.858  (0.109) | | | 0.858  (0.207) | | | 0.547  (0.439) | | 0.138  (0.381) | | | -0.038  (0.404) | | | 0.604  (0.371) | |
|  |  |  | Overweight | | 0.561  (0.406) | | | 0.648  (0.361) | | | 0.647  (0.287) | | | 0.794  (0.273) | | | -0.038  (0.362) | | -0.070  (0.456) | | | -0.093  (0.451) | | | 0.603  (0.345) | |
|  |  |  | Obese | | 0.544  (0.325) | | | 0.494  (0.329) | | | 0.459  (0.252) | | | 0.632  (0.313) | | | -0.082  (0.339) | | -0.132  (0.320) | | | -0.126  (0.324) | | | 0.229  (0.314) | |
| Pelvic obliquity | | | Normal | | 0.195  (0.513) | | | 0.187  (0.533) | | | 0.186  (0.478) | | | 0.020  (0.478) | | | 0.594  (0.335) | | 0.758  (0.208) | | | 0.588  (0.328) | | | 0.816  (0.278) | |
|  |  |  | Overweight | | 0.050  (0.451) | | | 0.014  (0.475) | | | 0.026  (0.346) | | | 0.001  (0.380) | | | 0.460  (0.297) | | 0.726  (0.181) | | | 0.601  (0.288) | | | 0.859  (0.120) | |
|  |  |  | Obese | | -0.044  (0.337) | | | -0.098  (0.312) | | | 0.178  (0.297) | | | -0.105  (0.393) | | | 0.366  (0.370) | | 0.646  (0.259) | | | 0.555  (0.258) | | | 0.657  (0.181) | |
| Pelvic rotation | | | Normal | | 0.456  (0.344) | | | 0.357  (0.356) | | | 0.264  (0.447) | | | 0.244  (0.437) | | | 0.776  (0.132) | | 0.454  (0.493) | | | 0.585  (0.534) | | | 0.894  (0.172) | |
|  |  |  | Overweight | | 0.346  (0.377) | | | 0.315  (0.428) | | | 0.308  (0.379) | | | 0.157  (0.471) | | | 0.692  (0.257) | | 0.432  (0.491) | | | 0.737  (0.309) | | | 0.876  (0.198) | |
|  |  |  | Obese | | 0.205  (0.452) | | | 0.065  (0.473) | | | 0.102  (0.434) | | | -0.098  (0.478) | | | 0.554  (0.262) | | 0.273  (0.407) | | | 0.700  (0.208) | | | 0.858  (0.153) | |

Above table summarised the obtained value from pelvic kinematic waveforms for normal, overweight and obese subjects using two different methods (CM= Cluster method and TM= Traditional method). The intra and inter session repeatability and variability of the kinematic data were investigated for all three planes (T=tilt, O=obliquity, R=rotation) using the CMC and standard deviation of the maximum pelvic tilt, obliquity and rotation for eight different activities of daily living.
